# Impact of in-school antigen testing on the prevalence of asymptomatic SARS-CoV-2 carriers during the COVID-19 pandemic era

**DOI:** 10.3389/fpubh.2026.1851915

**Published:** 2026-09-09

**Authors:** Christoph Zurl, Benoit Bernar, Sereina A. Herzog, Peter Willeit, Reinhold Kerbl, Thomas Müller, Bernd Lamprecht, Robert Krause, Michael Wagner, Volker Strenger, on behalf of the School-SARS-CoV-2 study group

**Affiliations:** 1 Division of General Pediatrics, Department of Pediatrics and Adolescent Medicine, Medical University of Graz, Graz, Austria; 2 Division of Infectious Diseases, Department of Internal Medicine, Medical University of Graz, Graz, Austria; 3 Research Unit for Host Pathogen Interaction, Medical University Graz, Graz, Austria; 4 BioTechMed-Graz, Graz, Austria; 5 Department of Pediatrics I, Medical University of Innsbruck, Innsbruck, Austria; 6 Institute for Medical Informatics, Statistics and Documentation, Medical University of Graz, Graz, Austria; 7 Institute of Clinical Epidemiology, Public Health, Health Economics, Medical Statistics and Informatics, Medical University of Innsbruck, Innsbruck, Austria; 8 Ignaz Semmelweis Institute, Interuniversity Institute for Infection Research, Vienna, Austria; 9 Department of Public Health and Primary Care, University of Cambridge, Cambridge, United Kingdom; 10 Department of Pediatrics and Adolescent Medicine, LKH Hochsteiermark, Leoben, Austria; 11 Department of Pulmonology, Faculty of Medicine, Kepler-University-Hospital, Johannes Kepler University Linz, Linz, Austria; 12 Vienna Covid-19 Detection Initiative, Vienna, Austria; 13 Department of Microbiology and Ecosystem Science, Centre for Microbiology and Environmental Systems Science, University of Vienna, Vienna, Austria; 14 Department of Chemistry and Bioscience, Center for Microbial Communities, Aalborg University, Aalborg, Denmark; 15 Division of Pediatric Pulmonology and Allergology, Department of Pediatrics and Adolescent Medicine, Medical University Graz, Graz, Austria

**Keywords:** COVID-19, children, school closure, school reopening, asymptomatic SARS-CoV-2 carriers, screening

## Abstract

**Introduction:**

In Austria, a nationwide antigen-test-based screening program for all students and educational staff in combination with intensified non-pharmaceutical mitigation measures were initiated in February 2021. We analyzed the first 6 weeks of this program and its impact on the prevalence of asymptomatic SARS-CoV-2 carriers.

**Methods:**

During the study period, we evaluated the results of 1 million students and approximately 150,000 educational staff members performing self-collected anterior-nasal swabs for antigen self-tests (ANAST) 1 to 3 times a week. Prevalence of asymptomatic SARS-CoV-2 carriers was monitored by an RT-qPCR based surveillance study in a representative school cohort.

**Results:**

During the 6 weeks period, 6.979 persons tested positive by ANAST, corresponding to a weekly positivity rate of median 0.08% (range 0.04–0.13) and 0.22% (range 0.12–0.30) for students and educational staff, respectively. Compared to students, staff members had a significantly higher chance of testing positive in all analyzed weeks with odds ratios (OR) up to 3.4 (95% CI: 3.0–3.8). Per week a total of 78.5–89.7% (median 84.1%) of positive ANASTs were reported as isolated cases. Evaluating the prevalence of asymptomatic SARS-CoV-2 carriers with a model adjusted for regional community incidence, the OR for having an RT-qPCR-detected SARS-CoV-2 infection after initiation of the screening program compared to earlier periods was 0.31 (95% CI 0.17–0.54; *p* < 0.001).

**Discussion:**

The screening program detected a considerable number of screening-positive individuals. Their prevalence was significantly lower during the screening program consistent with a potential contribution of in-school screening programs to reducing the spread of SARS-CoV-2 in educational settings.

## 1 Introduction

The COVID-19 pandemic caught the world largely unprepared and led to profound societal disruptions and widespread restrictions across the globe. In an effort to curb viral transmission, measures were implemented that affected nearly all areas of daily life. Many of these interventions were introduced in a context of considerable uncertainty and, due to the lack of prior experience and the limited availability of robust studies at the time, could not initially be grounded in a strong evidence base. Notably, the contribution of children and adolescents to transmission dynamics, and thus the potential risk associated with schools, was the focus of considerable debate. Therefore, many countries initially imposed school closures beside other measures to contain the pandemic (1) while mitigation strategies were shown to be able to reduce in-school transmission (2–6). Meanwhile, there are amounts of studies showing negative consequences of school closures (7–10), while its impact on the general and age specific incidence is still under discussion (11, 12). To safely re-open schools, screening programs were implemented in some regions (13). However, the specific effect of single mitigation measures in the educational setting remains unclear (14).

So far, several studies analyzed screening programs in educational setting. However, these studies are based on data modeling (14–17) or the reported screening programs were restricted to rather low numbers of voluntary participants or random samples in selected institutions, mostly comprised low frequency of testing and did not evaluate the epidemiological effects of these programs (12, 13, 18–23). In contrast, we performed repeated cross-sectional surveys to assess the uptake and number of positive cases after implementation of a nationwide, repeatedly performed surveillance program of ANAST in all students and educational staff, which was mandatory for school attendance and analyzed the changes of the overall prevalence in schools following the implementation of this testing strategy to evaluate a comprehensive and nationwide testing program in a real world setting.

## 2 Materials and methods

### 2.1 Observation period

Schools re-opened after general school closure and term break on February 8th (2 Austrian federal states) and 15th, 2021 (the remaining 7 federal states), respectively. We prospectively analyzed the results from the first weeks of a screening program from its start on February 15^th^, 2021, until Easter holidays starting on March 26th, 2021 corresponding to a homogenous 6-week late-winter period without general school closures. During the observation period, the national 7-day incidence in Austria increased from 110.4 to 243.7 cases per 100,000 inhabitants, coinciding with the transition toward Alpha predominance and the subsequent period during which Alpha became the dominant circulating SARS-CoV-2 variant. At that time, COVID-19 vaccination was available only for high-risk patients and health care workers. So, it can be assumed that neither educational staff members nor students were vaccinated to a meaningful extent at that time.

### 2.2 Setting in schools

From the beginning of the 2020/2021 school year, the following mitigating measures in schools were in place: reduced mixing of groups, regular ventilation of rooms, intensified hand hygiene, canceling of singing and use of wind instruments, canceling of indoor sports and facemasks at the discretion of school managements. Children with symptoms suggestive of SARS-CoV-2 infection were asked not to attend school. During the study period, mitigation measures were modified. In addition to the ANAST screening program, group sizes in secondary levels were reduced by cohorting into 2 groups (alternating school attendance on Monday/Tuesday or Wednesday/Thursday, distance learning for both groups on Friday). In contrast, students of primary schools continued to attend schools 5 days a week (as usual). Usage of facemasks became mandatory across all school districts (primary schools: surgical masks outside the classroom, secondary level I: surgical masks inside and outside the classroom, secondary level II: FFP-2/KN95 masks inside and outside the classroom).

### 2.3 Testing strategy

For school attendance, a negative result of a SARS-CoV-2 antigen rapid test was mandatory. In primary schools, students tested on Mondays and Wednesdays (and additionally on Fridays from March 19th on), students in the secondary levels tested once a week on the first of 2 days of school attendance (referred to as ANAST screening program). ANAST were performed in the morning prior to school start in the class rooms under supervision of educational staff. Students with positive ANAST were sent home immediately and reported as suspected SARS-CoV-2 cases to the local health authorities who initiated further evaluation by PCR testing. Educational staff were free to undergo testing at school or to provide proof of a negative test result obtained outside the school setting.

### 2.4 Anterior nasal antigen self-tests

For the ANAST, two different lateral flow device products were in use: Lepu Medical SARS-CoV-2 Antigen Rapid Test® (Lepu Medical Technology, Beijing, China, sensitivity reported by the manufacturer: 91.3%) for students of primary schools and secondary level I and the Flowflex SARS-CoV-2 Antigen Rapid Test® (ACON Biotech, Hangzhou, China, sensitivity reported by the manufacturer: 97.4%) for staff and students of secondary level II. Both tests were shown to have very high specificity >99.0% (24). According to the manufacturers´ manuals, swab was inserted 2–3 cm into the anterior nasal cavity and rotated 5 times on both sides. Further procedures were in accordance with usual lateral flow device procedures.

### 2.5 Data collection

Approximately 5,700 Austrian schools with around 1 million students aged 6 years and older and 150 thousands educational staff were included. For every school level (primary level, grade 1 to 4, age 6 to 9 years; secondary level I, grade 5 to 8, age 10 to 14 years; secondary level II, grades 9 to 12, age 15 to 19 years) aggregated data on students refusing testing, performed tests, number of tested students and number of positive tested students were reported weekly from every school to the regional school authorities of the respective federal state. Additionally, schools with two or more positive ANAST results in a class within a week were reported (referred to as class/schools with accumulation of cases following a definition of the Federal Ministry of Education, Science and Research). These data for each week, school level and federal state were collected by the Federal Ministry of Education, Science and Research and provided to the study team for further analysis.

According to general data protection regulation, no participant-level data but only aggregated data could be provided by schools and were available for analyses. Consequently, the following data were not available for analyses: number of positive ANAST which were falsified by PCR (false positive ANAST), number of infected students detected by PCR (e.g., due to symptoms or as a close contact) but priorly tested negative by ANAST (false negative), data on contact tracing and data on symptoms at the time of testing or in the further course.

In addition, data on weekly general and age-specific incidence of laboratory confirmed cases in the community were provided by the Austrian Agency for Health and Food Safety (AGES, in the following referred to as 7-day community incidence).

Furthermore, we included data obtained from the Austrian School-SARS-CoV-2 study (in the following referred to as PCR surveillance study), a nationwide prospective cohort study monitoring a representative sample of students at Austrian schools throughout the 2020/2021 school year (25). This study enabled quantification of the prevalence of asymptomatic SARS-CoV-2 infection in Austrian schools at different time points by repeatedly testing participants using a gargling solution and RT-qPCR. Further details on methods of this study and the results of the first 2 assessment periods (autumn 2020) have been described earlier (25). While the first 2 assessment periods of the aforementioned study were performed before the ANAST screening program was initiated, the third assessment period was performed simultaneously with the analyzed first weeks of the ANAST screening program (March 2021).

### 2.6 Data analyses

Positivity rates were calculated either as number of positive tests per test provided to staff and students or as positive tested individuals divided by the number of individuals participating in the screening program. Categorical values are presented as absolute numbers and percentages and continuous variables are presented as median and range or interquartile. For ANAST results, odds ratios (OR), i.e., chance of having a positive ANAST, are reported with exact 95% confidence interval (CI) by Fisher. For comparison of the prevalence of SARS-CoV-2 infection in students with and without the ANAST screening program, we adjusted the obtained prevalences for the corresponding regional community incidence. For the PCR surveillance study, ORs were estimated using mixed-effect logistic regression models regressing indicators for the time period (using period 1 as reference or – alternatively – both period 1 and 2 as reference group) and regional community incidence on the outcome of testing positive in the PCR test, with random intercepts at the participant level and resulting 95% CIs being calculated from robust standard errors estimated based on Sandwich estimators clustered at the school level.

A *p*-value < 0.05 was considered statistically significant. Statistical analyses were performed using R (Version 4.6.0)^1^ and Stata MP (Version 15.1, Stata Corp, Texas, US).

### 2.7 Ethical considerations

The main analysis of ANAST data is based on aggregated data obtained for legal and quality control reasons by the Federal Ministry of Education, Science and Research and the AGES. In these data, no individual-related data were included. Therefore, neither informed consent nor ethical approval was required for the analyses presented in this part of the study. However, the legal representatives of all students had to provide consent for participation in the screening program, which was mandatory for in-person school attendance. For the PCR surveillance study, ethics approvals were obtained by the ethics committees of the Medical University of Graz (no. 32–672 ex 19/20), Medical University of Innsbruck (no. 1319/2020), the Johannes Kepler University of Linz (no. 1222/2020), and the University of Vienna (no. 00591/2020).

For the PCR surveillance studies, written informed consent was obtained from all participants or their legal representative (25).

## 3 Results

### 3.1 Participation and provided tests

Approximately 1 million students and 150,000 educational staff members in Austria participated in the screening program, whereas per week a median 14.3 thousand students (1.42%, range 1.27–1.72) and 6.2 thousand educational staff (4.14%, range 2.82–6.30) denied participation in the screening program. Participation rates increased in students and educational staff from 98.28 and 95.07% in the first week to 98.73 and 97.18% in the last week of the analyzed period, respectively. Details on participants of different school levels, number of provided and positive ANAST as well as rate of participants with positive ANAST are presented in Table 1.

**Table 1:** Characteristics and results of the nationwide ANAST school screening program during the six-week observation period.

Participant group	Primary level	Secondary level I	Secondary level II	All schools
Registered at the beginning of the observation period [thousand]				
Students	360.9	319.7	325.9	1,006.6
Teacher	55.0	94.9		150.0
Non-participants per week [thousand] (median, range)				
Students	6.0 (4.8–7.2)	4.1 (3.9–6.2)	4.1 (2.8–4.5)	14.3 (12.8–17.3)
Teacher	1.3 (0.9–2.0)	4.9 (3.4–7.4)		6.2 (4.2–9.4)
Percentage of non-participants per week [%] (median, range)				
Students	1.65 (1.33–1.99)	1.28 (1.22–1.93)	1.25 (0.86–1.38)	1.42 (1.27–1.72)
Teacher	2.40 (1.60–3.67)	5.14 (3.53–7.83)		4.14 (2.82–6.30)
Participants per week [thousands] (median, range)				
Students	355.0 (353.8–356.1)	315.6 (313.5–315.8)	321.9 (321.4–323.1)	992.3 (989.3–993.7)
Teacher	53.7 (53.0–54.1)	90.1 (87.5–91.6)		143.7 (140.5–145.7)
Tests provided per week [thousands] (median, range)				
Students	679.1 (643.4–909.0)	388.8 (316.5–405.3)	310.5 (247.7–332.0)	1,389.1 (1,207.7 - 1,644.1)
Teacher	67.8 (54.4–93.3)	106.2 (74.9–117.5)		174.0 (129.3–210.8)
Positive tests in the observation period [n]				
Students	2,424	1,110	1,573	5,107
Teacher	947	925		1,872
Positive tests per week [n] (median, range)				
Students	326 (199–727)	181 (94–280)	278 (98–358)	842 (391–1,279)
Teacher	142 (105–227)	168 (65–197)		310 (170–424)
Percentage of persons with positive tests per week [%] (median, range)				
Students	0.09 (0.06–0.20)	0.06 (0.03–0.09)	0.09 (0.03–0.11)	0.08 (0.04–0.13)
Teacher	0.26 (0.20–0.43)	0.16 (0.09–0.20)		0.22 (0.12–0.30)
OR of positive tests (median, range)				
Students	1	1		1
Teacher	3.32 (1.11–4.55)	2.49 (1.83–3.48)		3.18 (1.43–3.36)

**Figure 1:**
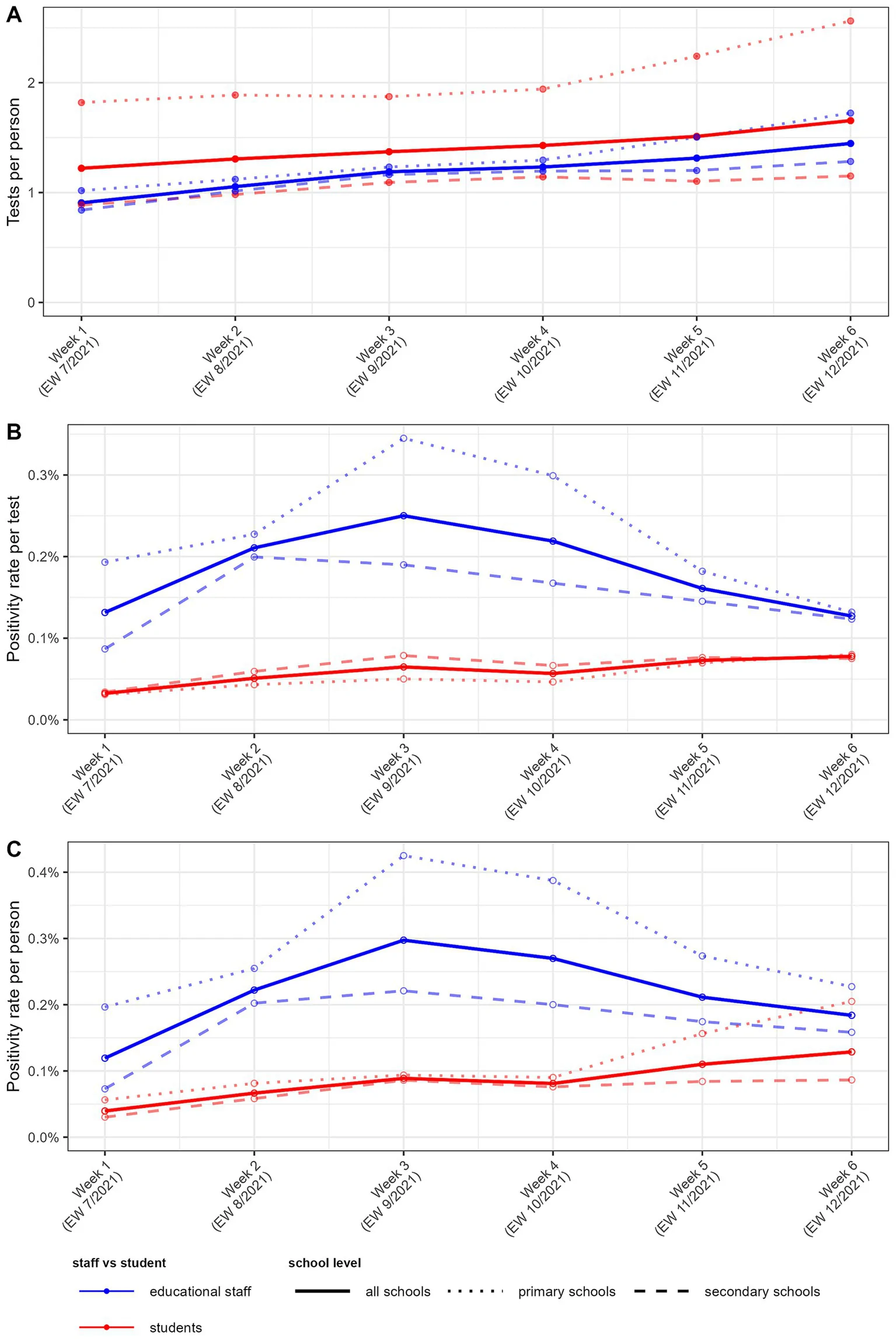
Number of provided tests per person **(A)**, rate of positive tests per provided test **(B)**, and rate of positive tested students/educational staff **(C)** on general and for each school level for every week: Educational staff (blue) and students (red); all schools (solid line), only primary schools (dotted line), and only secondary schools (dashed line). Week 1 is starting on Feb 15, 2021; the epidemiological week (EW) is stated in brackets.

The number of weekly provided tests per person increased from 1.22 to 1.65 in students and from 0.91 to 1.45 in educational staff. Due to more frequent testing in primary schools, students and staff in these schools had higher numbers of provided tests per person. This difference further increased during the last 2 weeks, when testing frequency was increased from 2 to 3 times per week whereas testing in secondary schools continued only once weekly (Figure 1A).

### 3.2 Positive-tested persons and changes over time

Over the entire course of the study, a total of 6,979 positive tests were reported (5,107 in students and 1,872 in educational staff, Table 1). Median positivity rate per week was 0.08% in students and 0.22% in educational staff. The rate of positive tested students increased from 0.04% in the first week to 0.13% in the last week (Figure 1C). In educational staff, rate of positive-tested individuals increased from 0.12% in the first week to a peak of 0.30% in the 3rd week and decreased, thereafter, to 0.18% (Figure 1C).

### 3.3 Differences between staff and students and between different school levels

**Figure 2:**
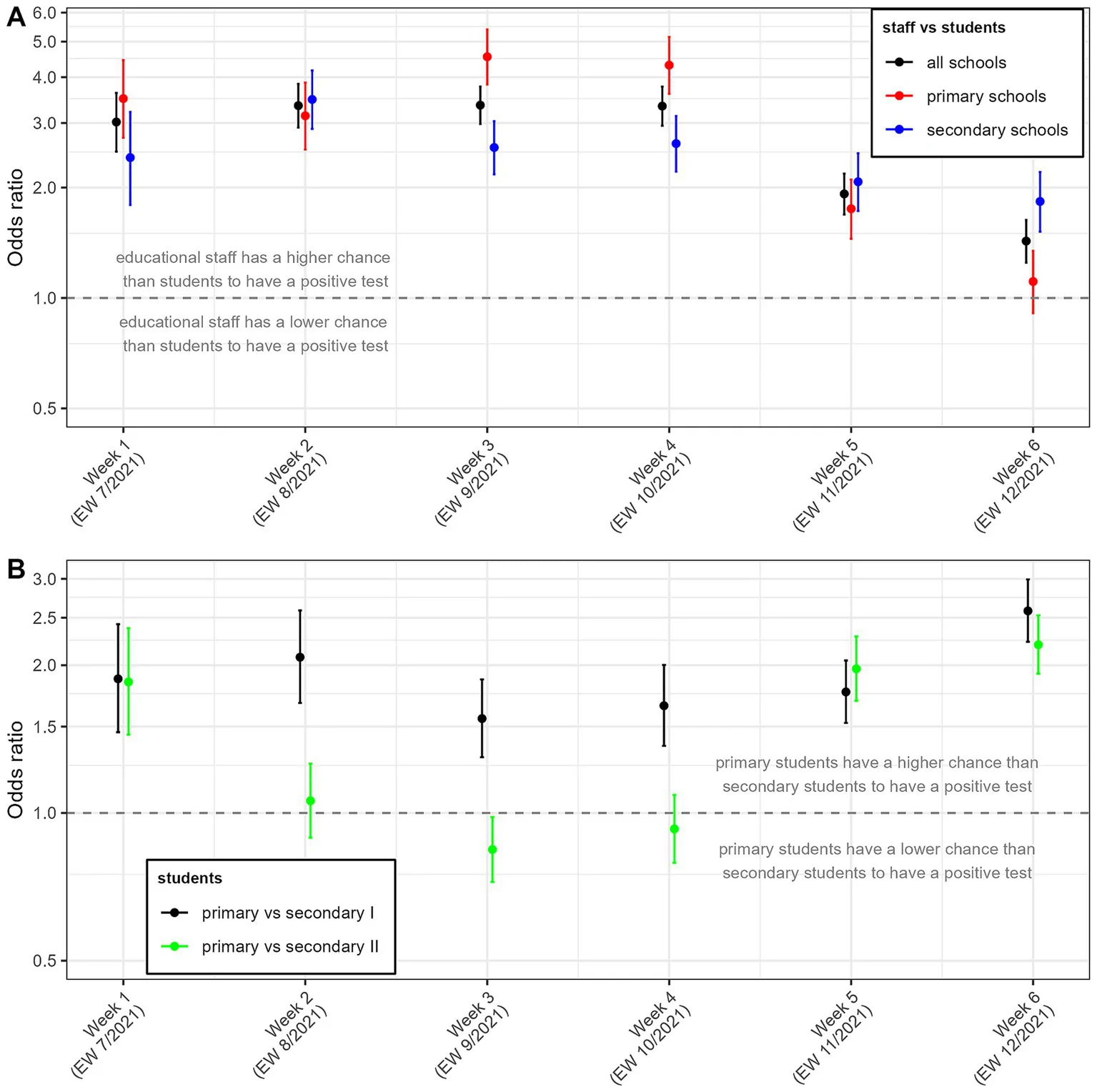
Odds ratio (OR) of having a positive test result being an educational staff compared to a student, overall **(A)** and by school type **(A)**, i.e.: OR > 1 means that educational staff has a higher chance than students to have a positive test. OR of having a positive test result being a primary student compared to a secondary student **(B)**, i.e.: OR > 1 means that primary student has a higher chance than a secondary students to have a positive test. The observed decrease in weeks 5 and 6 is mainly due to the decrease in OR at primary school level. Note that students at primary school level were tested 3 times per week in weeks 5 and 6 compared to twice a week in weeks 1–4 (Figure 1A). However, also the positivity rate per tests increased in those 2 weeks (Figure 1B). Week 1 is starting on Feb 15, 2021; the epidemiological week (EW) is stated in brackets.

The chance of having a positive ANAST was higher for the educational staff compared to students in all analyzed weeks with OR up to 3.4 (95% CI: 3.0–3.8, Figure 2A). Interestingly, there were differences in OR between teachers of primary and secondary schools in weeks 3 and 4 (higher OR in primary schools) and week 6 (higher OR in secondary schools, Figure 2A).

The likelihood of having a positive ANAST was higher for primary school students compared to students of secondary level I in all analyzed weeks. ORs of having a positive ANAST for students of primary level compared to students of secondary level II were heterogeneous over the weeks (Figure 2B).

### 3.4 Comparison with general age-specific incidence of laboratory confirmed cases

**Figure 3:**
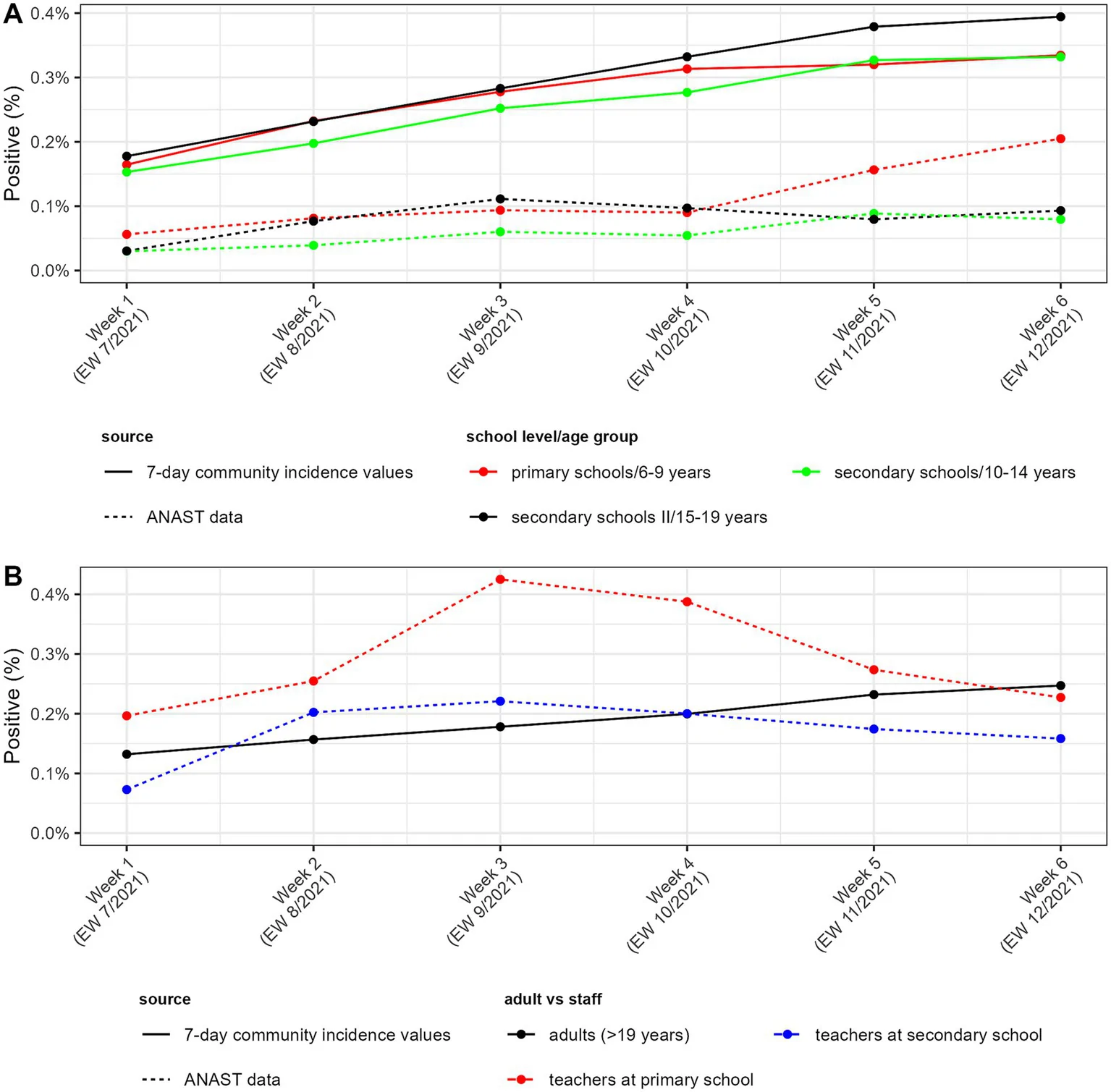
Rate of students and educational staff tested positive with ANAST (i.e., positive tested persons per week/tested persons per week, dashed lines) compared to national data on 7-day community incidence of laboratory confirmed cases (solid lines); for students compared with corresponding age group **(A)**, and educational staff compared with adult data (>19 years, **B**). While the rate of students tested positive by ANAST increased in parallel with the general incidence of the respective age groups during the observation period **(A)**, rate of positive tested educational staff showed a steeper increase in the first 3 weeks and a likewise steep decline thereafter. This was more pronounced in educational staff of primary schools **(B)**. The observed increase of positive ANAST results in primary levels students in weeks 5 and 6 can be explained by an increase of the test frequency. There was no increase in positivity rates per test in primary level students (see Figure 1). Week 1 is starting on Feb 15, 2021; the epidemiological week (EW) is stated in brackets.

We compared positivity rate of ANAST in educational staff and students of different school levels and weeks with the weekly and general and age-specific 7-day community incidence reported to the national health authorities (Figure 3). In contrast to educational staff members, the rate of students tested positive by ANAST increased in parallel with the general incidence of the respective age groups during the observation period.

### 3.5 Schools with accumulated cases

During the observation period, 274 events with accumulated cases (defined as schools with two or more positive ANAST results in a class within a week) including 978 students in 211 different schools were reported with increasing numbers from 24 schools (0.4%) in week 1 to 43 (0.8% of 5,700 schools) in week 6. In the affected schools, 2 to 64 (IQR 2–5, median 3) students and 1 to 6 (median 1) classes were affected, respectively. However, in one primary school reporting 62 positive tests, all positive tests were confirmed to be false positive by PCR. In this school, all tests of 3 packages (originating from 1 lot) gave false positive results while the tests from other packages of the same lot showed no conspicuous results. For the analyses, these results proven to be false positive were excluded.

Per week 78.5 to 89.7% (median 84.1%) of positive ANASTs were reported as isolated cases without other concurrent positive cases in the same class.

### 3.6 Change of prevalence of SARS-CoV-2 infection in schools

During the 2020/2021 school year, we evaluated a representative sample of students at different time periods for prevalence of SARS-CoV-2 infection detected with RT-qPCR (25). Positivity rate in the PCR surveillance study was higher than in the ANAST screening program (median 0.21% vs. 0.11%). In a model adjusted for regional community incidence, the OR for having an RT-qPCR-detected SARS-CoV-2 infection in March 2021 (with ANAST screening in place) compared to earlier assessment periods of the study (28 September to 16 November 2020, no ANAST screening in place) was 0.31 (95% CI 0.17–0.54; *p* < 0.001). The corresponding ORs by school type were 0.43 (95% CI 0.22–0.86; *p* = 0.017) in primary schools and 0.14 (95% CI 0.05–0.43; *p* < 0.001) in lower secondary schools, which were not significantly different from each other (P for interaction = 0.085; Table 2).

**Table 2:** SARS-CoV-2 prevalence among students before and during implementation of the nationwide ANAST school screening program and odds ratios adjusted for regional community incidence.

Characteristic	Period 1	Period 2	Period 3
	Prior to ANAST screening program		concurrent with ANAST screening program
Assessment period	28/09/2020 to 22/10/2020	10/11/2020 to 16/11/2020	01/03/2021 to 18/03/2021
Median regional 7-day community incidence/100,000 inhabitants (IQR)^a^	76 (43–124)	410 (385–640)	185 (141–225)
No. of participating schools	243	88	244
No. of study participants	10,156	3,745	7,523
No. of RT-qPCR detected SARS-CoV-2 cases	40	52	16
Period prevalence (95% CI)^b^	0.39% (0.28–0.55%)	1.39% (1.04–1.85%)	0.21% (0.13–0.36%)
Odds ratio for SARS-CoV-2 (95% CI) adjusted for regional community incidence^a^^,^^b^^,^^c^			
Compared to period 1	[Reference]	0.86 (0.41–1.81) [*p* = 0.692]	0.29 (0.15–0.54) [*p* < 0.001]
Compared to period 1 and 2	[Reference]		0.32 (0.18–0.56) [*p* < 0.001]

a Community incidence data were obtained from the AGES dashboard on 16/08/2021 and were merged to schools on the district level. Median and IQR values are calculated for the regional 7-day community incidence values across schools—for period 1, 2 and 3, separately.

b 95% CI were calculated from robust standard errors estimated based on Sandwich estimators clustered at the school level.

c Odds ratios were estimated using mixed-effect logistic regression models with random intercepts at the participant level.

## 4 Discussion

In this study, we describe a nationwide screening program for SARS-CoV-2 based on anterior nasal antigen self-tests performed 1 to 3 times per week by all Austrian students and educational staff. Participation was voluntary, yet mandatory for school attendance. Acceptance of the program was high, with a participation rate clearly over 98% that increased over time. During the first 6 weeks of this program, 5,000 (0.51%) of approximately 1 million Austrian students as well as 1,900 (1.25%) of 150,000 educational staff tested positive by ANAST and were consequently isolated and evaluated by RT-qPCR. Contact tracing and isolation of close contacts of the positive-tested students and educational staff helped in additionally detecting, minimizing and probably preventing further transmissions. Therefore, like other screening programs (26) this in-school screening program should have also contributed to mitigate transmission in the community.

Positivity rate of ANAST in teachers was higher than in students. There are some possible explanations for this observation: (i) the incidence of SARS-CoV-2 infection might be higher in teachers, (ii) ANAST might be more effectively performed by teachers, (iii) sensitivity of ANAST might be lower in asymptomatic children and adolescents due to a possibly lower general viral load in this age group, (iv) children’s viral load might be specifically lower in the anterior-nasal cavity, or (v) the time of viral shedding is shorter in children (27) and, therefore, the probability of detection might be lower. With higher detection rates in adults, it seems feasible to have educational staff included in screening programs.

Students in primary schools tested positive more frequently than students in secondary schools. This finding may partly be explained by differences in testing frequency, as primary school students were tested two to three times per week while attending school 5 days per week, whereas secondary school students attended school only 2 days per week and were therefore tested only once weekly. However, given that SARS-CoV-2 infections generally remain detectable for several days, students tested once weekly would still be expected to remain test-positive at the time of screening in a substantial proportion of cases. On the other hand, the differing extent of in-person school attendance may itself have contributed to differences in infection risk. Finally, different antigen tests were used in different participant groups, with the Lepu assay used in primary and lower secondary levels (Secondary I) and the Flowflex assay used in upper secondary level (Secondary II) and educational staff. Although the reported sensitivity of the Flowflex assay was higher than that of the Lepu assay, higher positivity rates were observed both in educational staff (Flowflex) compared with students and in primary school students (Lepu) compared with secondary school students (Flowflex). This suggests that differences in test performance alone are unlikely to fully explain the observed findings. Nevertheless, the use of different test platforms represents a potential source of residual confounding.

Despite the well-known sensitivity limitations of antigen-based tests (24, 28), two observations support that the ANAST screening program in conjunction with other intensified mitigation measures reduced SARS-CoV-2 prevalence in schools. First, after correcting for differences in the community incidence, prevalence of asymptomatic SARS-CoV-2 carriers detected by the PCR surveillance study was more than two thirds lower in March 2021 than in autumn 2020 (25). Second, after week 3 of the screening program, we observed a decreasing or at least stable incidence of positive ANAST results in secondary levels students and in educational staff, despite a simultaneous rise in the national incidence of reported laboratory confirmed cases of SARS-CoV-2 infections. This observation may reflect a preventive effect of the implemented mitigation measures, including the screening program, but should not be interpreted as evidence of causality.

Apart from the ANAST screening program performed in all school levels, group size reduction and general mask wearing was another mainstay of the intensified mitigation measures exclusively applied in secondary levels. However, reduction of the prevalence of asymptomatic SARS-CoV-2 carriers was likewise seen in primary level students without significant differences compared to secondary level students.

Furthermore, the dynamics of different circulating SARS-CoV-2 variants (Alpha, B.1.1.7; Beta, B.1.351; Gamma, P.1), seasonal and behavioral factors (including weather conditions and indoor congregation) and changes in isolation policies may have influenced not only transmissibility but also disease manifestation, including the proportion of asymptomatic versus symptomatic infections. Such temporal changes may have affected the observed prevalence independently of the screening program and therefore represent a potential source of residual confounding. However, the analyses demonstrating a decreasing prevalence were adjusted for local incidence, thereby accounting for differences in overall community transmission. While this adjustment does not fully account for potential temporal changes in the proportion of asymptomatic versus symptomatic infections, it reduces confounding related to changes in overall community transmission. Therefore, changes in variant circulation and disease presentation alone are unlikely to fully explain the observed decline in prevalence.

The antigen tests used for self-collected anterior-nasal swabs have much lower sensitivities compared to RT-qPCR (24, 28). The higher positivity rate observed in the contemporaneous RT-qPCR surveillance study (0.21% vs. 0.11% in ANAST) indicates that additional asymptomatic infections were detected despite the nationwide screening program, reflecting the lower sensitivity of antigen testing and representing an important limitation of the screening approach. On the other hand, antigen tests are cheap, easy to use and provide results within a few minutes allowing immediate isolation of positive-tested persons. Antigen-based screening programs were discussed controversially (29–31) as testing was performed in large cohorts with relatively low prevalence. Therefore, following a positive antigen test, RT-qPCR is needed to confirm or rule out SARS-CoV-2 infection. Still, as testing was considered an essential element in the pandemic, routine testing of students opened the opportunity to test a wide range of individuals that otherwise may not have participated in other testing programs. Infected individuals not detected by ANAST might have lower viral load and therefore might show lower infectivity. This hypothesis is in line with studies showing sensitivity of SARS-CoV-2 antigen tests are depending on CT values (as a surrogate of viral load) (28) and the fact that individuals with lower viral load show lower infectivity (32). Finally, despite a relatively high specificity of ANAST reported previously (24), positive and negative predictive values and therefore the usefulness of every mass screening strongly dependent on disease prevalence, with a reduction in the positive predictive values due to false positives when prevalence is very low.

While single infected students and educational staff reflect infections imported from the community into the schools, clusters including several students within classes rather indicate in-school transmission. In the analyzed 6 weeks, 274 events of accumulated cases including 978 students from 211 schools were detected by the ANAST screening program. While these cases comprise 19% of all screening-positive cases, the remaining 81% were detected as single cases. While only 3.4% of schools reported accumulated screening-positive cases, the vast majority of schools did not. This might indicate a minor impact of in-school transmission. As individual linkage to confirmatory PCR results was not available, screening-positive cases cannot be equated with PCR-confirmed infections. Consequently, accumulated screening-positive cases within classes should be interpreted with caution, as some may have resulted from false-positive antigen test results despite the high specificity of the assay.

Of note, in one primary school, a cluster of 62 positive tests (all tests obtained from 3 packages) turned out to be false-positive by consecutive PCR testing. Unexpected results like this indicate methodical problems (e.g., defective tests due to incorrect storage) and should be interpreted with caution.

Beyond epidemiological effectiveness, school-based screening programs may also have psychosocial consequences, including distress or potential stigmatization of students testing positive. These aspects were not evaluated in the present study and should be considered in future evaluations of large-scale school screening programs.

### 4.1 Limitations

According to general data protection regulation, only aggregated data were available for analyses. Consequently, individual-level data on confirmatory RT-qPCR results viral sequencing, subsequent contact tracing or the development of symptoms in screening-positive individuals were not available. The effects of mitigation measures (which especially were intensified in the secondary levels) cannot be quantified and clearly distinguished from the effects of the screening program. Due to the lower sensitivity of the ANAST compared to RT-qPCR (24) the data do not allow a reliable description of the real prevalence of asymptomatic carriers, which on the other hand was evaluated by the PCR surveillance study. Furthermore, our data originate from a period of shifting SARS-CoV-2 variant circulation and prior to the predominance of more infectious variants of concern. Our findings might not apply for more contagious variants or other viruses in future pandemics.

In conclusion, this program was able to detect a substantial number of infected and probably infective individuals. The vast majority was reported as isolated cases without other concurrent positive cases in the same class indicating low in-school transmission under the mitigation measures implemented. Despite high general and age-specific incidence of reported RT-qPCR-confirmed SARS-CoV-2 infections, the prevalence of asymptomatic SARS-CoV-2 infections among students remained substantially lower than expected based on comparable periods without the ANAST screening program. These findings are consistent with a beneficial contribution of the screening program, together with the accompanying mitigation measures, to limiting SARS-CoV-2 transmission, although a causal effect cannot be established from the present data.

## Data Availability

The datasets presented in this article are not readily available because complexity. Requests to access the datasets should be directed to volker.strenger@medunigraz.at.

## Footnotes

1
www.r-project.org

## Group memEbers of the school-SARS-CoV-2 study group

Andrea Berghold, Buck Hanson, Evelyn Stelzl, Heribert Stoiber, Johannes Zuber, Robert Heinen, Alwin Köhler, David Bernhard, Wegene Borena, Christian Doppler, Dorothee von Laer, Hannes Schmidt, Johannes Pröll, Ivo Steinmetz, and Mariam Al-Rawi.
